# An Advanced Tool Wear Forecasting Technique with Uncertainty Quantification Using Bayesian Inference and Support Vector Regression

**DOI:** 10.3390/s24113394

**Published:** 2024-05-24

**Authors:** Zhiming Rong, Yuxiong Li, Li Wu, Chong Zhang, Jialin Li

**Affiliations:** 1 Applied Technology College, Dalian Ocean University, Dalian 116023, China; rongzhiming@dlou.edu.cn; 2 School of Mechanical Engineering, Dalian Jiaotong University, Dalian 116028, China; djtuwuli@163.com (L.W.); zcsyl@djtu.edu.cn (C.Z.); 3 Chongqing Engineering Laboratory for Transportation Engineering Application Robot, Chongqing Jiaotong University, Chongqing 400074, China; jialinli_neu@163.com

**Keywords:** cutting tool wear prediction, brownian motion, bayesian inference, uncertainty quantification, support vector regression

## Abstract

Tool wear prediction is of great significance in industrial production. Current tool wear prediction methods mainly rely on the indirect estimation of machine learning, which focuses more on estimating the current tool wear state and lacks effective quantification of random uncertainty factors. To overcome these shortcomings, this paper proposes a novel method for predicting cutting tool wear. In the offline phase, the multiple degradation features were modeled using the Brownian motion stochastic process and a SVR model was trained for mapping the features and the tool wear values. In the online phase, the Bayesian inference was used to update the random parameters of the feature degradation model, and the future trend of the features was estimated using simulation samples. The estimation results were input into the SVR model to achieve in-advance prediction of the cutting tool wear in the form of distribution densities. An experimental tool wear dataset was used to verify the effectiveness of the proposed method. The results demonstrate that the method shows superiority in prediction accuracy and stability.

## 1. Introduction

Machine tools play a critical role in the development of modern manufacturing. Various industries, including automotive manufacturing, aerospace, and military industries, rely heavily on the precision-processed components produced by machine tools [1,2,3]. The efficient and accurate processing capability of machine tools is indispensable. During the machining process, the cutting tool, as a key component that directly contacts the workpiece, is directly related to the precision of the product and the stability of the processing. Nevertheless, cutting tools are bound to wear out due to intense vibration and thermal coupling effects [4,5]. The damaged cutting tool may not only lead to a decrease in machining accuracy, but also cause safety accidents, bringing a threat to the operators. Furthermore, the severe wear status of cutting tools can cause a decrease in processing efficiency and increased downtime for the machine, which seriously affect the entire production line [6,7]. If the wear status can be obtained during the machining process, enabling the timely maintenance and replacement of cutting tools, the efficiency and safety of the manufacturing can be further improved. Therefore, estimation and prediction methods of cutting tool wear have been developed.

The mainstream methods for cutting tool wear estimation and prediction can be generally classified into two categories: direct methods and indirect methods [6]. Direct methods can obtain the current tool wear status by directly observing the quality or shape variation of the cutting tools [8,9]. Optical imaging is one of the most common direct methods for cutting tool wear monitoring. It uses the phenomenon that the worn surface has a higher reflectivity than that of the unworn surface [10,11]. This characteristic is utilized to obtain the wear values of the cutting tools. The advantage of direct methods is that they can visually describe the health status of the cutting tools, thus obtaining the accurate measurement of the tool wear [12]. However, due to the intense vibration generated in the machining process, direct methods are only effective when the machine tools are stopped, thus they cannot achieve a continuous online observation [6,12].

With the rapid development of signal acquisition and information technology, indirect methods have gradually become the hot spot in cutting tool wear prediction. Indirect methods establish machine learning models to analyze the mapping relationship between the cutting tool wear and the sensor signals, including vibration signals, cutting force, acoustic emission signals, and so on [13]. Compared with direct methods, indirect monitoring is more flexible in its application and does not interfere with the machining process of the machine tool, making it possible to achieve online real-time cutting tool wear estimation and prediction [14].

The artificial neural network (ANN) is a basic machine learning tool that is commonly used in cutting tool wear prediction [6]. Wang et al. [15] proposed a novel tool wear prediction, combining the physical model of the cutting tools and the ANN algorithms. The physical model was also used to construct the loss function and explore the information from unlabeled samples. Xu et al. [16] established a multi-scale convolutional gated recurrent unit network, in which the multi-scale CNN model was used to effectively extract features from the raw sensor signals and the recurrent unit network model was trained to learn the relationship between the features and the flank tool wear. Qin et al. [17] used unsupervised K-means clustering to adaptively classify the wear stage and constructed the self-coding network to reduce the complexity of the degradation features; therefore, improving the efficiency of the calculation. Cheng et al. [18] proposed a firefly algorithm-optimized back propagation (BP) neural network for tool wear prediction, which can effectively select the hyperparameters of the network, thus enhancing the prediction performance. Wang et al. [19] presented an improved neural network model for tool wear estimation, with a Siamese structure and an auxiliary input to enhance the feature extraction ability. Benefiting from the excellent nonlinear mapping processing capabilities of ANN, the ANN-based models have achieved satisfying results in cutting tool wear prediction. Some other machine learning algorithms have also been applied in tool wear prediction, such as support vector machines (SVM) [20,21], fuzzy inference [22,23], and so on.

To further improve the processing capability of nonlinear mapping and thereby improve the accuracy of tool wear prediction, some researchers have proposed prediction models based on deep learning algorithms. Wang et al. [24] developed a novel deep heterogeneous gated recurrent unit (GRU) model, using expertise and information to enhance the feature learning ability and building an intermediate layer to analyze the relationship between input and output. Shah et al. [25] combined the Walsh–Hadamard transforming and deep convolutional generative adversarial network (DCGAN) to effectively select and extract the degradation features, so as to increase the prediction accuracy of the tool wear. Liu et al. [26] developed an intelligent tool wear monitoring system, containing a multi-input parallel convolutional network to select and preprocess the multi-scale degradation features, and a long short-term memory (LSTM) model was established to achieve the estimation of the tool wear. Abdeltawab et al. [27] divided the tool wear status into five stages and established a CNN-LSTM model to achieve the online identification of wear stage. 

Overall, the existing machine learning-based tool wear prediction methods have been widely applied in engineering practice and have achieved satisfactory prediction results. With the accurate tool wear estimation at the current time, it is possible to determine the maintenance and replacement strategy, thereby improving the safety and efficiency of the machining process. However, the existing methods still have certain shortcomings that require further enhancement: First, the machine learning-based prediction methods typically require the input of currently observed signals and output wear prediction results at the current time, thus lacking the ability to predict future wear trends. If the wear status of the cutting tools can be predicted in advance, it will bring tremendous assistance to the maintenance and replacement of the cutting tools. Second, the existing methods lack the quantification of uncertainty. The degradation signals generated in the machining process generally contain large amount of uncertainties, such as random noises and individual variations, which lead to significant fluctuations in the tool wear prediction results. Although the filtering algorithms can address the uncertain factors and enhance the stability of the prediction, the final results are still presented in the form of deterministic values. If the uncertainties can be comprehensively considered and quantified into the final prediction results, the persuasiveness of the prediction can be enhanced. 

Based on these two issues, this paper presents a novel cutting tool wear prediction method, which fully considers the uncertainties, and can achieve an in-advance prediction of wear. The main contribution of the paper can be summarized as follows: (1) The Brownian motion stochastic process was used to establish the degradation models for multiple degradation features; (2) The model parameters were updated using Bayesian inference and the future trend of features was estimated in the form of random simulation samples considering the uncertainties; (3) An SVR model was established to describe the relationship between the degradation features and the cutting tool wear, and the future estimations of the features were input to obtain the future wear trend; (4) A degradation dataset of the cutting tools was introduced to test the effectiveness of the proposed method.

The rest of the paper is organized as follows: Section 2 presents the overall process of the proposed tool wear prediction method; Section 3 illustrates the degradation modeling of multiple features, as well as the parameter updates using Bayesian inference; Section 4 presents the prediction of future trends of features using random sampling and introduces the SVR modelling for tool wear online estimation and the future prediction of tool wear, fully considering the uncertainties; In Section 5, a degradation dataset of cutting tools is used as a numerical example, to test the effectiveness of the presented work; Section 6 provides a summary of the paper and a discussion of the effectiveness.

## 2. Comprehensive Flow of the Method

**Figure 1:**
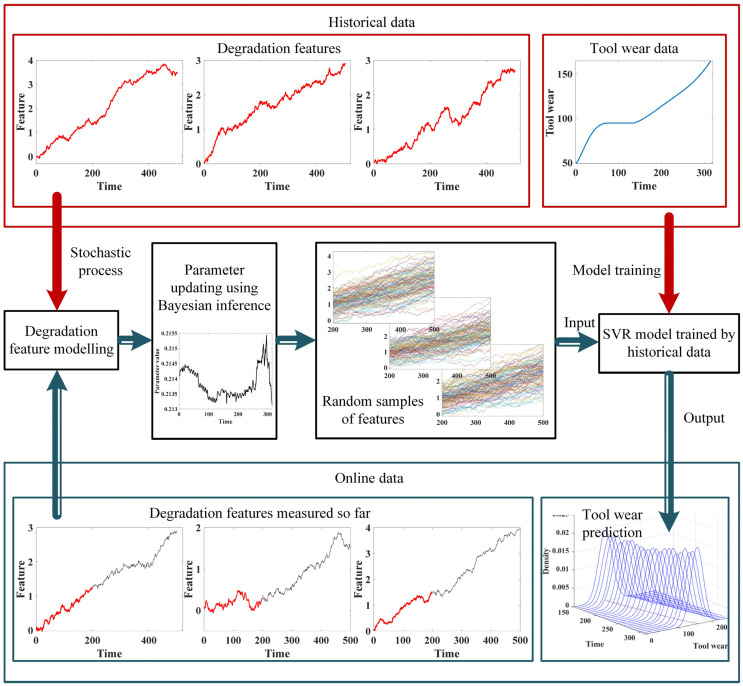
Procedure of the proposed method.

This paper presents a novel tool wear prediction method, combining the Brownian motion stochastic process, Bayesian inference, and an SVR model. The proposed method is capable of quantifying the uncertainties in the machining process, generating the tool wear prediction in the form of probability, distribution, and forecasting the future wear variation trend. The overall process of the method is summarized below, and the specific procedure is illustrated in Figure 1:

Offline phase:

Step 1: Modelling for degradation features

Utilize the Brownian motion stochastic process to develop degradation models that capture the temporal evolution of multiple time–frequency features.

Step 2: SVR training

Utilize historical data to train an SVR model that maps the degradation features to the corresponding trends in cutting tool wear. This model will serve as a predictive tool for estimating future wear based on observed degradation features.

Online phase:

Step 3: Parameter updates

Utilize Bayesian inference techniques to update the parameters of the degradation models based on newly observed online data of the multiple degradation features.

Step 4: Random simulation for future trend estimation

With the updated parameters, utilize random simulation samples to estimate the future trends of the multiple degradation features.

Step 5: Tool wear prediction

Input the estimated future values of the degradation features, obtained from the random simulations, into the pre-trained SVR model. The output of the SVR model in probability distribution is achieved, which quantifies the likelihood of different levels of cutting tool wear in the near future.

## 3. Degradation Feature Modelling Based on the Stochastic Process

During the machining process, sensors can be installed to capture various signals that are closely related to cutting wear, including the cutting forces acoustic emission signal. By extracting time–domain features from these signals, multiple degradation features, which can intuitively reflect the degradation process of the cutting tools, can be obtained. To quantify the uncertainties in the machining process, the degradation features were modeled using the stochastic process. During the online prediction phase, based on the real-time observed data, the model parameters were updated using Bayesian inference.

### 3.1. Brownian Motion Stochastic Process

Brownian motion is a stochastic process model with continuous time parameters and continuous states, which has critical application value in theoretical mathematics and modern engineering. In the degradation process of equipment and components, the degradation indicators generally show strong non-monotonicity, due to the random noises and environment interference. The mathematical properties of Brownian motion can effectively adapt to and fit the non-monotonic phenomena in the degradation process, further improving the accuracy of modeling.

Suppose that *D*(*t*) is the value of the degradation features at the time *t*, the Brownian motion stochastic process for degradation modeling can be expressed as follows:

(1) $$D\left(t\right)=D\left(0\right)+\theta \phi \left(t,\lambda \right)+\sigma B\left(t\right)$$

where *θ* represents the Gaussian-distributed random parameter that is used to describe the individual variation in same type of component or equipment; *λ* is a fixed parameter that represents the similarity between the same type of component or equipment; *θϕ*(*t*, *λ*) is the drift term of the degradation, which characterizes the degradation pattern of the component; *B*(*t*) is the standard Brownian motion and *σ* is the diffusion coefficient. The term *σB*(*t*) is used to characterize the uncertainties in the degradation process. 

**Figure 2:**
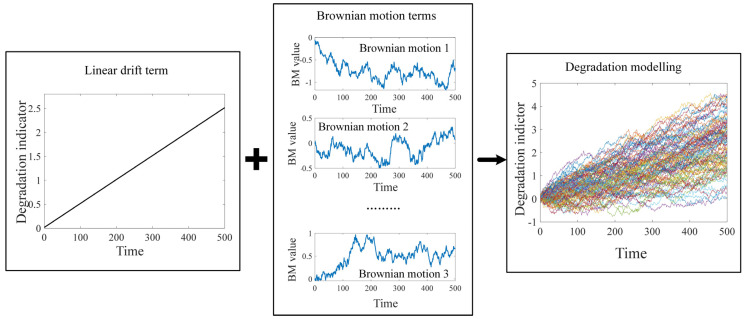
Degradation modelling using the Brownian motion stochastic process.

The standard Brownian motion is a continuous stochastic process with Markov properties, and its increments follow independent Gaussian distributions. The use of standard Brownian motion (BM) allows for the full consideration of uncertainties, as well as the non-monotonicity of degradation in the establishment of degradation models; thus, the model can significantly reflect the actual degradation situation of the mechanical equipment or components in practical scenarios. Furthermore, the drift term can capture various degradation patterns using linear or nonlinear expression, significantly enhancing the applicability of the model. Figure 2 shows the simulation and modelling using the Brownian motion stochastic process. It can be seen that the degradation models can effectively reflect the actual degradation process; therefore, the health condition assessment and remaining life prediction can be furthered facilitated. 

### 3.2. Bayesian Parameter Updates

During the machining process, the sensors installed on the machine tool continuously capture various signals, such as cutting forces and vibration signals; therefore, the Bayesian inference method can be used to fully utilize the online monitored data and update the parameters of the degradation model, achieving more accurate prediction results for tool wear or remaining life. The basic principle of the Bayesian parameter updates is to derive the posterior distribution of the model parameters based on the prior distribution and online monitoring data from the sensors, using the Bayesian formula, as Equation (2) [28]:

(2) $$p\left(\theta |D\right)=\frac{p\left(D|\theta \right)p\left(\theta \right)}{p\left(D\right)}\propto p\left(D|\theta \right)p\left(\theta \right)$$

where *p*(*θ|D*) is the posterior distribution of random parameter *θ*, representing a more precise and specific estimation combining the prior distribution and online observed data; *p*(*θ*) is the prior distribution of *θ*, representing the empirical data of the parameter; *p*(*D*|*θ*) is the likelihood function, which represents the probability of the observed data occurring under certain parameter values. 

Since the increments of Brownian motion follow the independent Gaussian distributions, the individual increments of the degradation feature *D*, given the values of *θ* and *λ* from *t*_1_ to *t_k_*, also follow the independent Gaussian distributions, as expressed in Equation (3):

(3) $$p\left(\Delta D_{k}|\theta \right)=\frac{1}{\sqrt{2\pi \sigma^{2}\left(t_{k}-t_{k-1}\right)}}\exp\left[-\frac{{\left(\Delta D_{k}-\theta \left(\phi \left(t_{k},\lambda \right)-\phi \left(t_{k-1},\lambda \right)\right)\right)}^{2}}{2\sigma^{2}\left(t_{k}-t_{k-1}\right)}\right]$$

Suppose that ***D***_1:*k*_ = [*D*(*t*_1_), *D*(*t*_2_), …, *D*(*t_k_*)] represents the observed degradation features from time *t*_1_ to *t_k_*, the likelihood function *p*(***D***_1:*k*_|*θ*) can be expressed in the form of the joint density function, as shown in Equation (4):

(4) $$p\left(D_{1:k}|\theta \right)=\prod_{i=1}^{k}\frac{1}{\sqrt{2\pi \sigma^{2}\left(t_{i}-t_{i-1}\right)}}\exp\left[-\frac{{\left(D\left(t_{i}\right)-D\left(t_{i-1}\right)-\theta \left(\phi \left(t_{i},\lambda \right)-\phi \left(t_{i},\lambda \right)\right)\right)}^{2}}{2\sigma^{2}\left(t_{i}-t_{i-1}\right)}\right]$$

This property of the conjugate probability distributions ensures that the prior and posterior distributions belong to the same family of probability distributions in Bayesian inference. Based on this, the prior of parameter *θ* is set as a Gaussian distribution, i.e., $\theta \sim N\left(\mu_{\theta ,0},\sigma_{\theta ,0}^{2}\right)$. Therefore, the following equations can be obtained:

(5) $$p\left(\theta |D_{1:k}\right)=\frac{1}{\sqrt{2\pi \sigma_{\theta ,k}^{2}}}\exp\left[-\frac{{\left(\theta -\mu_{\theta ,k}\right)}^{2}}{2\sigma_{\theta ,k}^{2}}\right]$$

(6) $$\begin{array}{c} p\left(D_{1:k}|\theta \right)p\left(\theta \right)=\frac{1}{\sqrt{2\pi \sigma_{\theta ,0}^{2}}}\exp\left[-\frac{{\left(\theta -\mu_{\theta ,0}\right)}^{2}}{2\sigma_{\theta ,0}^{2}}\right]\cdot \\ \;\;\;\;\;\;\;\;\;\;\;\;\;\;\;\;\;\;\;\;\;\;\;\;\;\;\prod_{i=1}^{k}\frac{1}{\sqrt{2\pi \sigma^{2}\left(t_{i}-t_{i-1}\right)}}\exp\left[-\frac{{\left(D\left(t_{i}\right)-D\left(t_{i-1}\right)-\theta \left(\phi \left(t_{i},\lambda \right)-\phi \left(t_{i},\lambda \right)\right)\right)}^{2}}{2\sigma^{2}\left(t_{i}-t_{i-1}\right)}\right] \end{array}$$

where $\mu_{\theta ,k}$ and $\sigma_{\theta ,k}^{2}$ are the posterior expectation and variance of *θ*. Based on the Bayesian formula, i.e., the posterior distribution is in direct proportion to the product of the prior distribution function and the likelihood function, indicating a positive correlation between them. Thus, the constant terms in Equations (5) and (6) can be eliminated. By setting the coefficients of the terms containing *θ*, the relationship between the prior parameters ($\mu_{\theta ,0},\sigma_{\theta ,0}^{2}$) and the posterior parameters ($\mu_{\theta ,k},\sigma_{\theta ,k}^{2}$) can be obtained, as follows [29]:

(7) $$\mu_{\theta ,k}=\frac{\mu_{\theta ,0}\sigma^{2}+\sigma_{\theta ,0}^{2}\sum_{i=1}^{k}\frac{\left(D\left(t_{i}\right)-D\left(t_{i-1}\right)\right)\left(\phi \left(t_{i},\lambda \right)-\phi \left(t_{i-1},\lambda \right)\right)}{\left(t_{i}-t_{i-1}\right)}}{\sigma^{2}+\sigma_{\theta ,0}^{2}\sum_{i=1}^{k}\frac{{\left(\phi \left(t_{i},\lambda \right)-\phi \left(t_{i-1},\lambda \right)\right)}^{2}}{\left(t_{i}-t_{i-1}\right)}}$$

(8) $$\sigma_{\theta ,k}^{2}=\frac{\sigma_{\theta ,0}^{2}\sigma^{2}}{\sigma^{2}+\sigma_{\theta ,0}^{2}\sum_{i=1}^{k}\frac{{\left(\phi \left(t_{i},\lambda \right)-\phi \left(t_{i-1},\lambda \right)\right)}^{2}}{\left(t_{i}-t_{i-1}\right)}}$$

As indicated by Equations (7) and (8), at the current time *t_k_*, based on the available observed values of the degradation features ***D***_1:*k*_, the online update of the posterior distribution can be achieved using Bayesian inference, which further improves the accuracy and applicability of the degradation model.

## 4. Tool Wear Prediction with Uncertainty Quantification

In this section, based on the updated model parameters from the previous section, a tool wear prediction method quantifying the uncertainties is proposed, combining the random sampling and the SVR model. The proposed method aims to achieve an in-advance estimation of the cutting tool wear in the form of a probability distribution, thus enhancing the stability of the prediction.

### 4.1. Degradation Feature Prediction Using Random Sampling

At time *t_k_*, as the posterior distribution of the model parameters has been achieved, the future degradation features can be predicted based on the distribution parameters and the properties of the Gaussian distributed increments in Brownian motion. Given the value of parameter *θ*, the increment of the degradation feature at time *t_k_* can be expressed as:

(9) $$D\left(t_{k+1}\right)-D\left(t_{k}\right)=\theta \left(\phi \left(t_{k+1},\lambda \right)-\phi \left(t_{k+1},\lambda \right)\right)+\sigma^{2}\left(B\left(t_{k+1}\right)-B\left(t_{k+1}\right)\right)$$

Since the posterior expectation of the random parameter *θ* has been obtained, the increment can be regarded as a random value following a Gaussian distribution as follows:

(10) $$D\left(t_{k+1}\right)-D\left(t_{k}\right)\sim N\left(\mu_{\theta ,k}\left(\phi \left(t_{k+1},\lambda \right)-\phi \left(t_{k},\lambda \right)\right),\sigma_{\theta ,k}^{2}{\left(\phi \left(t_{k+1},\lambda \right)-\phi \left(t_{k},\lambda \right)\right)}^{2}+\sigma^{2}\left(t_{k+1}-t_{k}\right)\right)$$

Therefore, the probability distribution of the degradation feature at *t_k_*_+1_ can be expressed in Gaussian distribution, as Equation (11):

(11) $$\begin{array}{c} p\left(D\left(t_{k+1}\right)\right)=\frac{1}{\sqrt{2\pi \left(\sigma_{\theta ,k}^{2}{\left(\phi \left(t_{k+1},\lambda \right)-\phi \left(t_{k},\lambda \right)\right)}^{2}+\sigma^{2}\left(t_{k+1}-t_{k}\right)\right)}}\cdot \\ \;\;\;\;\;\;\;\;\;\;\;\;\;\;\;\;\;\;\;\;\;\;\;\;\;\;\;\;\;\exp\left[-\frac{{\left(D\left(t_{k+1}\right)-D\left(t_{k}\right)-\mu_{\theta ,k}\left(\phi \left(t_{k+1},\lambda \right)-\phi \left(t_{k},\lambda \right)\right)\right)}^{2}}{2\left(\sigma_{\theta ,k}^{2}{\left(\phi \left(t_{k+1},\lambda \right)-\phi \left(t_{k},\lambda \right)\right)}^{2}+\sigma^{2}\left(t_{k+1}-t_{k}\right)\right)}\right] \end{array}$$

Similarly, the distribution of the degradation feature at time *t_k_*_+*l*_ can also be achieved, which is expressed in Equation (12):

(12) $$\begin{array}{c} p\left(D\left(t_{k+l}\right)\right)=\frac{1}{\sqrt{2\pi \left(\sigma_{\theta ,k}^{2}{\left(\phi \left(t_{k+l},\lambda \right)-\phi \left(t_{k},\lambda \right)\right)}^{2}+\sigma^{2}\left(t_{k+l}-t_{k}\right)\right)}}\cdot \\ \;\;\;\;\;\;\;\;\;\;\;\;\;\;\;\;\;\;\;\;\;\;\;\;\;\;\;\;\;\exp\left[-\frac{{\left(D\left(t_{k+l}\right)-D\left(t_{k}\right)-\mu_{\theta ,k}\left(\phi \left(t_{k+l},\lambda \right)-\phi \left(t_{k},\lambda \right)\right)\right)}^{2}}{2\left(\sigma_{\theta ,k}^{2}{\left(\phi \left(t_{k+l},\lambda \right)-\phi \left(t_{k},\lambda \right)\right)}^{2}+\sigma^{2}\left(t_{k+l}-t_{k}\right)\right)}\right] \end{array}$$

Based on Equation (12), the future degradation features can be estimated by generating a certain number of random samples, instead of obtaining fixed values, thereby effectively quantifying the uncertainties in the machining process and enhancing the credibility of the prediction. 

### 4.2. SVR Model for Tool Wear Prediction

SVR is an effective machine learning tool, widely used in small sample cases and multi-dimensional nonlinear mapping problems. Based on the SVR model, this section establishes a mapping model to describe the relationship between the multi-dimensional degradation features and the tool wear. 

Assume that the historical cutting tool degradation data $Z=\left\{x\in {\mathbb{R}}^{M\times N},y\in {\mathbb{R}}^{M\times 1}\right\}$ are available, where $x=\left[x_{1},x_{2},\cdots ,x_{M}\right]$ represents the *N*-dimensional degradation features with *M* time notes, and $y=\left[y_{1},y_{2},\cdots ,y_{M}\right]$ represents the corresponding tool wear values. To establish the mapping relationship between the degradation features and the tool wear, the following regression was constructed:

(13) $$f\left(x\right)=w\cdot \Phi \left(x\right)+b$$

where *w* and *b* represent the weight vector and intercept, respectively. $\Phi \left(x\right)$ represents the transformation from the n-dimensional real vector space to the higher-dimensional Hilbert space. The ultimate goal of the SVR modelling is to achieve the appropriate values of *w* and *b*. The basic principle of the SVR model is to establish a hyperplane to make the deviation between the regression values and the data samples less than a predetermined error, while ensuring that as many samples as possible are within the limited bounds of the hyper plane. The slack variables are also introduced to describe the acceptable degree of deviation for the samples; therefore, the regression problem is transformed into a optimization problem, as Equation (14) [30,31]:

(14) $$\begin{array}{c} \min\;\;l\left(w,\xi ,\xi^{*}\right)=\frac{1}{2}w^{T}w+C\sum_{i=1}^{M}\left(\xi_{i}+\xi_{i}^{*}\right)\\ s.t.\left\{\begin{array}{c} y_{i}-\left[w\phi \left(x\right)+b\right]\leq e+\xi_{i}\\ \left[w\phi \left(x\right)+b\right]-y_{i}\leq e+\xi_{i}^{*}\\ \xi_{i},\xi_{i}^{*}\geq 0 \end{array}\right. \end{array}$$

where *e* represents the loss factor, indicating the maximum tolerable error; ξ is the slack factor, describing the tolerance for outliers in the sample; *C* is the penalty coefficient, representing the penalty for incorrect samples. To simplify the optimization problem, Lagrange multipliers *α* are introduced, incorporating complex constraints into the optimization objective function; thus, the optimization problem is transformed into the following form [30,31]:

(15) $$\begin{array}{c} \max\;\;l_{\alpha }\left(\alpha ,\alpha^{*}\right)=-\frac{1}{2}\sum_{i,j=1}^{M}\left(\alpha_{i}^{*}-\alpha_{i}\right)\left(\alpha_{j}^{*}-\alpha_{j}\right)\Phi \left(x_{i}\right)\Phi \left(x_{j}\right)\\ -\sum_{i=1}^{M}e\left(\alpha_{i}+\alpha_{i}^{*}\right)+\sum_{i=1}^{M}y_{i}\left(\alpha_{i}^{*}-\alpha_{i}\right)\\ s.t.\left\{\begin{array}{c} \sum_{i=1}^{M}\left(\alpha_{i}-\alpha_{i}^{*}\right)=0\\ 0\leq \alpha_{i},\alpha_{i}^{*}\leq C \end{array}\right. \end{array}$$

By inputting the randomly sampled degraded features obtained in Section 4.1 into the established SVR model, the prediction for future tool wear with quantified uncertainties can be obtained, in the form of probability distributions.

## 5. Experimental Study

To verify the effectiveness of the proposed cutting tool wear prediction method, an application based on the PHM 2010 cutting tool degradation dataset [32] was constructed. Some of the current tool wear estimation methods were also introduced to test the superiority of the proposed method. 

### 5.1. Information of the Tool Wear Dataset

The experiment was conducted using a high-speed RFM760 CNC milling. The dataset contained six subsets, with each recording the degradation signals, including cutting forces, vibration signals, and the acoustic emission of the milling cutters, from multiple sensors. During the experiment, six tungsten carbide ball-end milling cutters were used to perform the cutting operations on the HRC52 stainless steel workpiece. The same cutting parameters were adopted throughout the entire machining process, as shown in Table 1.

The Kistler piezo accelerometers were installed on the workpiece to collect the vibration signals of the cutting tools during the machining process, in x, y, and z directions. A Kistler quartz three-component platform dynamometer was installed to sensor the cutting forces signals during the machining process. The collected data of the following sensors were conditioned through corresponding signal accessories and the voltage data were collected using a NI DAQ PCI 1200 board. The cutter’s flank wear was measured after each cut using a LEICA MZ12 microscopy system, and there were a total of approximately 300 individual data acquisition files (one for each cut) [32]. Thus, the time units of this experimental case were defined as “cutting times”.

**Figure 3:**
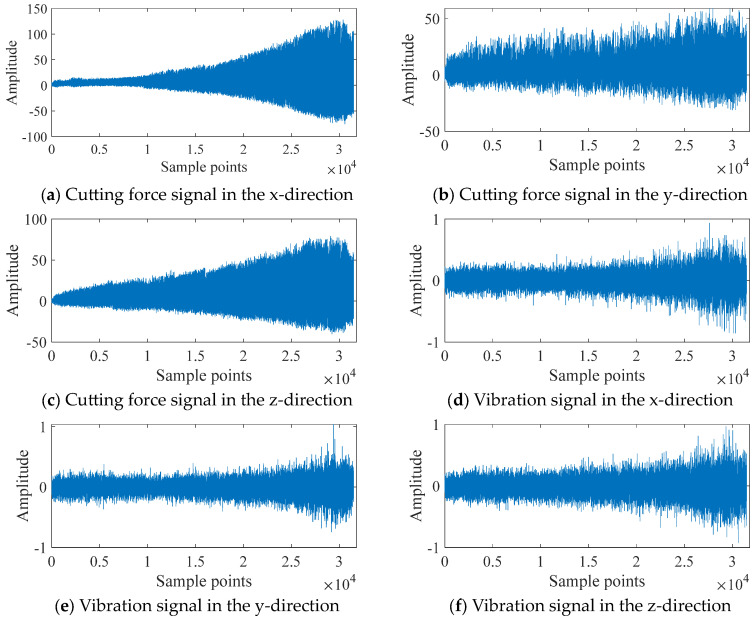
Original signals for C4.

Figure 3 depicts the x-direction cutting force signal, the y-direction cutting force signal, the z-direction cutting force signal, the x-direction vibration signal, the y-direction vibration signal, the z-direction vibration signal, and the acoustic emission signal of tool C4 after undergoing a 1000:1 downsampling process.

### 5.2. Degradation Modelling of Multiple Features

**Figure 4:**
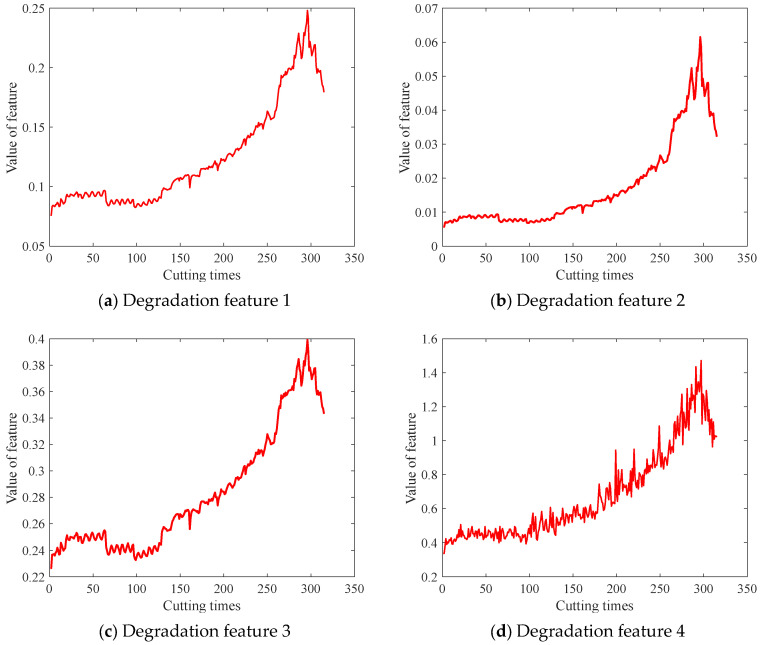

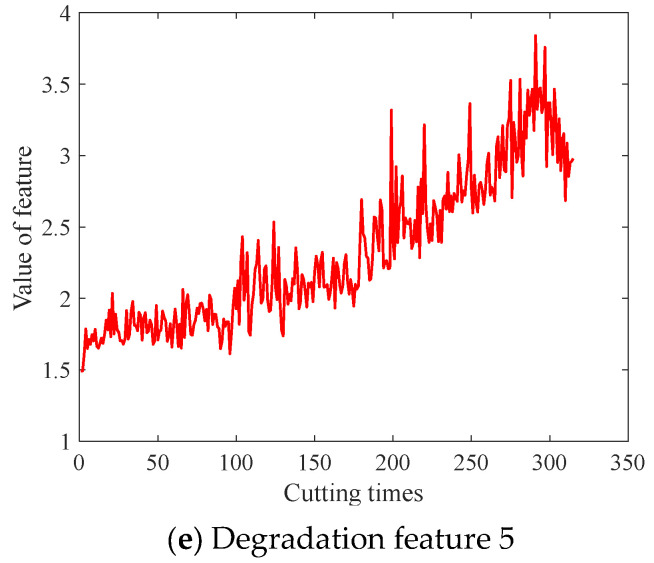
Degradation feature of the cutters C4.

In this paper, the subset C4 was used as the historical dataset to train the SVR model and estimate the initial parameters of the stochastic process. The subset C1 was used as the test set to verify the effectiveness of the proposed method. To fully reflect the degradation of the milling cutters, multiple degradation features were selected, as shown in Table 2. The selected features showed a distinct trend of change, thus enabling us to describe the degradation process of the cutters, as shown in Figure 4. These features were used as the input of the SVR model, and the Brownian motion stochastic process was applied for the degradation modelling. 

In the degradation modelling, the exponential function was chosen as the drift term of the stochastic process, i.e., *ϕ*(*t*, *λ*) = exp(*λt*). This is because the exponential function can effectively capture the nonlinearity and accelerated degradation characteristics of the degradation process. The initial parameters of the degradation models for multiple features were determined based on the historical data of C4, as shown in Table 3. 

**Figure 5:**
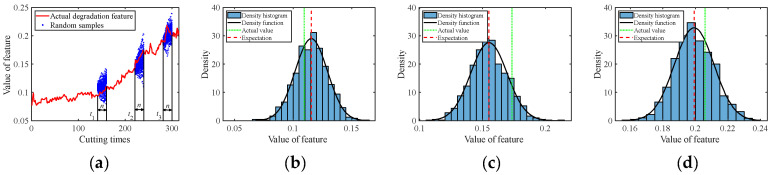
Prediction of degradation feature 1: (**a**) Random samples; (**b**) Distribution at time 160; (**c**) Distribution at time 240; (**d**) Distribution at time 300.

**Figure 6:**
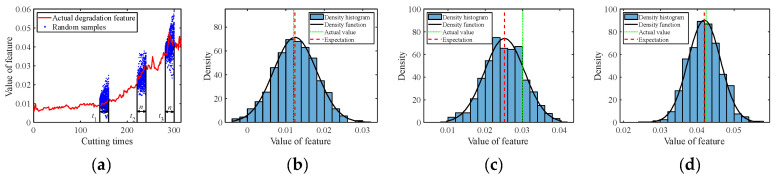
Prediction of degradation feature 2: (**a**) Random samples; (**b**) Distribution at time 160; (**c**) Distribution at time 240; (**d**) Distribution at time 300.

**Figure 7:**
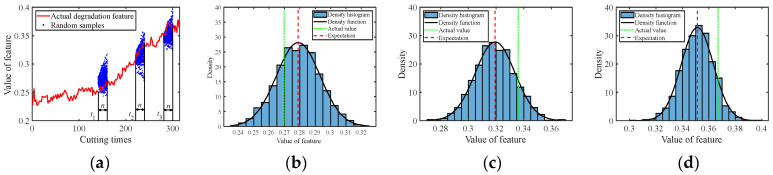
Prediction of degradation feature 3: (**a**) Random samples; (**b**) Distribution at time 160; (**c**) Distribution at time 240; (**d**) Distribution at time 300.

**Figure 8:**
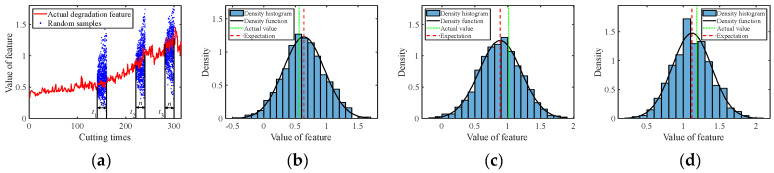
Prediction of degradation feature 4: (**a**) Random samples; (**b**) Distribution at time 160; (**c**) Distribution at time 240; (**d**) Distribution at time 300.

**Figure 9:**
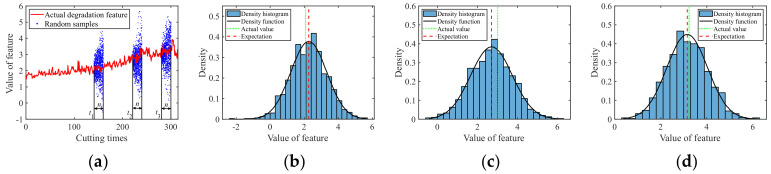
Prediction of degradation feature 5: (**a**) Random samples; (**b**) Distribution at time 160; (**c**) Distribution at time 240; (**d**) Distribution at time 300.

The C1 subset was used as the real-time observed degradation dataset during the online prediction phase. The degradation models for multiple degradation features were established based on Equation (1). At time *t_k_*, the model parameter $\mu_{\theta ,k}$ of each degradation feature was updated using the Bayesian formula, i.e., Equations (7) and (8), based on the online monitored data. After obtaining the latest parameters at time *t_k_*, random sampling was conducted for future time points according to Equation (12) to acquire the predicted feature values with uncertainty quantification, in the form of probability density. Figure 5, Figure 6, Figure 7, Figure 8 and Figure 9 show the prediction results of the multiple degradation features with random samples at several time points.

### 5.3. Wear Prediction Using the SVR Model

To establish the mapping relationship between the multiple degradation features and the tool wear, a Support Vector Regression (SVR) model was implemented, with the five features depicted in Figure 4 serving as the input variables and the corresponding tool wear as the output. The SVR model was trained using the data from C4, and the hyper parameters were optimized using Particle Swarm Optimization (PSO). It should be noted that the input degradation features did not have to undergo denoising, normalization, and other preprocessing measures; therefore, the information of the noises and other uncertainties remained. By inputting the predicted degradation feature values obtained in Section 5.2 into the trained SVR model, a series of tool wear prediction samples were generated, presenting the predicted results of the tool wear in the form of probability density.

**Figure 10:**
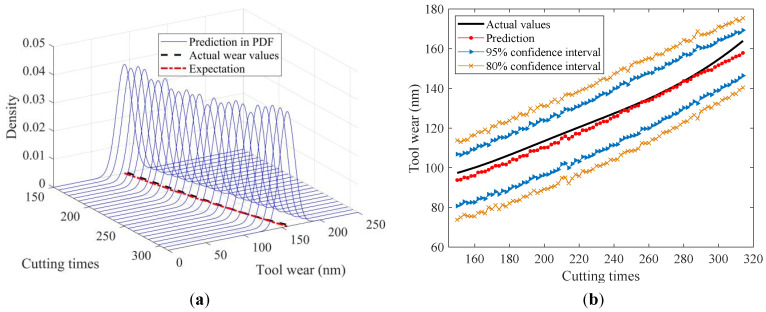
In-advance tool wear prediction for one time point ahead: (**a**) Probability density expression of tool wear at each time point; (**b**) Predicted expectation and confidence intervals.

**Figure 11:**
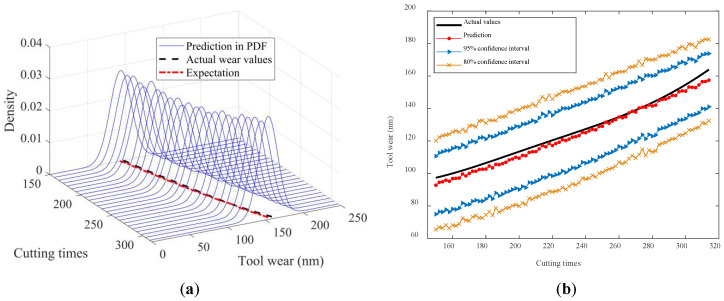
In-advance tool wear prediction for two time points ahead: (**a**) Probability density expression of tool wear at each time point; (**b**) Predicted expectation and confidence intervals.

**Figure 12:**
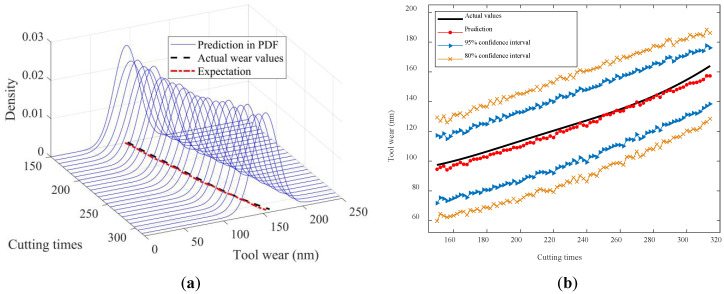
In-advance tool wear prediction for two time points ahead: (**a**) Probability density expression of tool wear at each time point; (**b**) Predicted expectation and confidence intervals.

**Figure 13:**
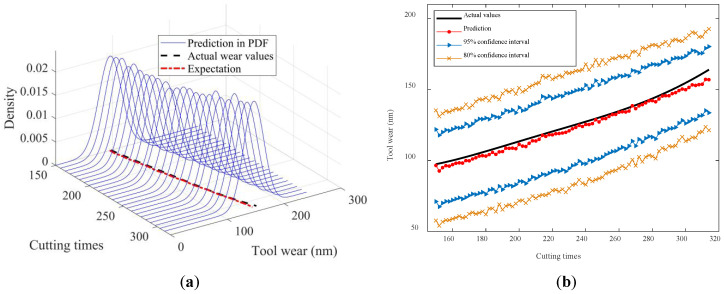
In-advance tool wear prediction for three time points ahead: (**a**) Probability density expression of tool wear at each time point; (**b**) Predicted expectation and confidence intervals.

**Figure 14:**
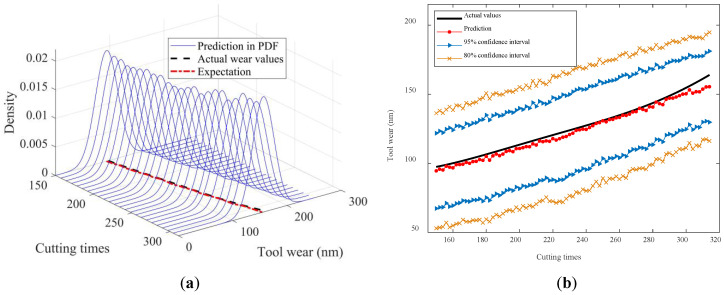
In advance tool wear prediction for four time points ahead: (**a**) Probability density expression of tool wear at each time point; (**b**) Predicted expectation and confidence intervals.

To visually illustrate the prediction results, Figure 10, Figure 11, Figure 12, Figure 13 and Figure 14 show the estimated tool wear prediction after the time point 150 (i.e., after 150 cutting times). These figures demonstrate the forecasted estimations for one to five time points ahead, respectively, obtained using the proposed method. These predictions not only capture the trend of the tool wear but also represent the uncertainty in the predictions through probability density, providing comprehensive and insightful information for assessing the state of the tool wear. Such predictions are crucial for guiding tool use and maintenance, enhancing production efficiency, and reducing operational costs.

The figures above clearly demonstrate that the proposed method was capable of achieving advance wear prediction with a high level of accuracy. At the current time point, the method precisely forecasted the potential wear condition of the cutting tool after five cutting times, closely aligning with the actual changes in the tool wear values. Moreover, the method was able to produce a prediction result in the form of a probability distribution with relevant confidence intervals. The proposed method effectively accounted for the uncertainties in the machining process, making the predictions more compelling and reliable. The probability distribution not only reflected the most likely wear state but also captured the range of possible outcomes, thus providing a more comprehensive view of the tool’s condition. The availability of confidence intervals further enables decision-makers to develop more suitable and rational strategies for tool replacement and maintenance. With a clear understanding of the potential wear range, decision-makers can plan ahead, minimizing the risk of unexpected tool failures and maximizing the utilization of each tool. This not only improves the safety of the machining process but also enhances its efficiency by reducing the unnecessary downtime for tool changes.

### 5.4. Comparison

**Figure 15:**
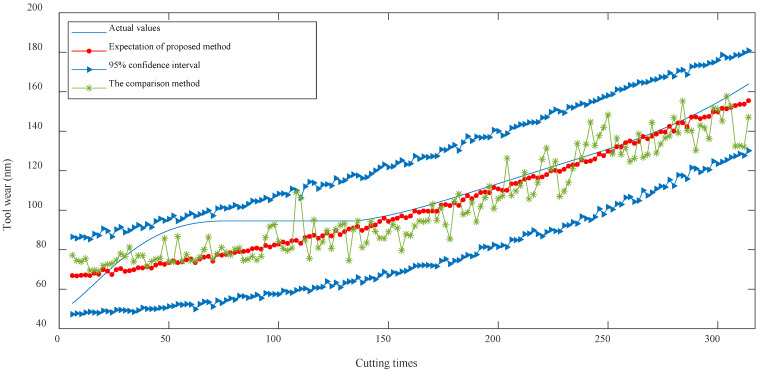
Comparison of prediction accuracy and stability between different methods.

In order to demonstrate the superiority of the proposed method, an SVR model was trained as a comparison, with the multiple time–domain degradation features. The degradation features were pre-processed using denoising and normalization, and the hyperparameters of the SVR model were optimized using a PSO algorithm. The results of each method are shown in Figure 15. As can be seen from the figure, the expectation of the tool wear prediction obtained using the proposed method was closer to the actual observed wear values, with smaller fluctuations and errors compared with the comparison method, thus resulting in better prediction stability. In addition, the comparison method can only provide a fixed predicted value at each time point, while the proposed method is capable of obtaining a confidence interval for the wear amount, making it more flexible and convenient. Table 4 shows the calculation of multiple prediction errors, further highlighting the advantages of the proposed method in prediction precision and stability compared with the other tool wear estimation method.

In order to verify the efficiency of the algorithm, the algorithm duration in both the offline training phase and the online prediction phase was calculated for the method proposed in this paper and the comparison method. As shown in Table 5, the results indicate that there was a relatively small difference in the time consumption for the model training between the method proposed in this paper and the comparison method, meaning that it did not sacrifice excessive computational efficiency to improve prediction accuracy. In the online prediction, due to the adoption of the random sampling method in this paper, the time consumption was higher than that of traditional methods. Nonetheless, the average prediction time required at each time node was only around 0.1 s, thus fully enabling efficient real-time prediction.

## 6. Conclusions

In this paper, a novel cutting tool wear prediction method combining the Bayesian inference and SVR models is proposed. The main innovation of the proposed method was to achieve an in-advance wear prediction, fully considering the uncertainties in the machining process. The main work of the paper is outlined as follows: (1) Using the Brownian motion stochastic process, the degradation models of features were established. The models contained the random parameters to estimate the uncertainties in the degradation; (2) The model parameters were updated during the online prediction phase, based on Bayesian inference. With the updated parameters, the future trends of the features were estimated by generating random samples; (3) The relationship between the features and the tool wear was analyzed by training the SVR model, and the random samples of the degradation features were input into the SVR model to achieve the prediction of the cutting tool wear; (4) To validate the effectiveness of this proposed method, a degradation dataset of cutting tools was utilized.

Compared with other methods, the method presented in this paper shows two distinct advantages. Firstly, it enables the advance estimation of tool wear, which means that it can predict the future wear conditions based on the current measured data. Secondly, the method proposed can fully consider the uncertainties in the machining process and output the prediction results in the form of probability distribution, which is beneficial for formulating maintenance and replacement plans for cutting tools. The experimental study in this paper also demonstrated the superiority of the method in prediction accuracy and stability.

## Figures and Tables

**Table 1:** Experimental parameter settings.

Spindle Speed	Feed Rate	Radial Cutting Depth (*y*-Axis)	Axial Cutting Depth (*z*-Axis)
10,400 rpm	1555 mm/min	0.125 mm	0.2 mm

**Table 2:** Selected degradation features.

Number	Feature	Calculation Formula
1	RMS of vibration (z-direction)	$D^{\left(1\right)}=\sqrt{\frac{1}{n}\sum_{i=1}^{n}x_{i}^{2}}$
2	Variance of vibration (z-direction)	$D^{\left(2\right)}=\frac{1}{n}\sum_{i=1}^{n}{\left(x_{i}-mean(x)\right)}^{2}$
3	Root square amplitude of vibration (z-direction)	$D^{\left(3\right)}={\left(\frac{1}{n}\sum_{i=1}^{n}\sqrt{\left|x_{i}\right|}\right)}^{2}$
4	Peak of vibration (z-direction)	$D^{\left(4\right)}=\max\left(\left|x\right|\right)$
5	Margin of vibration (z-direction)	$D^{\left(5\right)}=\frac{\max\left(\left|x\right|\right)}{{\left(\frac{1}{n}\sum_{i=1}^{n}\sqrt{\left|x_{i}\right|}\right)}^{2}}$

Note: *n* represents the total amount of data collected within a certain sampling segment

**Table 3:** Initial parameters of the degradation models.

Feature	Parameters	Value
RMS of vibration (z-direction)	$\mu_{\theta ,0}$	6.395 × 10^−2^
	b	3.802 × 10^−3^
	$\sigma^{2}$	1.048 × 10^−5^
Variance of vibration (z-direction)	$\mu_{\theta ,0}$	3.098 × 10^−3^
	b	8.811 × 10^−3^
	$\sigma^{2}$	1.552 × 10^−6^
Root square amplitude of vibration (z-direction)	$\mu_{\theta ,0}$	2.141 × 10^−1^
	b	1.679 × 10^−3^
	$\sigma^{2}$	1.078 × 10^−5^
Peak of vibration (z-direction)	$\mu_{\theta ,0}$	3.175 × 10^−1^
	b	4.285 × 10^−3^
	$\sigma^{2}$	5.450 × 10^−3^
Margin of vibration (z-direction)	$\mu_{\theta ,0}$	1.567 × 10^0^
	b	2.326 × 10^−3^
	$\sigma^{2}$	5.499 × 10^−2^

**Table 4:** Prediction errors.

	Proposed Method	Comparison SVR
Variance	40.9147	97.5408
RMSE	72.3540	135.6722
MRE	0.0686	0.0968
Correlation coefficient	0.9712	0.9231

**Table 5:** Computational load comparison.

Method	Training Time (s)	Total Online Prediction Time (s)	Average Online Prediction Time (s)
Proposed model	61.3696	35.1706	0.1117
Comparison SVR	97.2654	0.0509	0.0001

## Data Availability

Data are contained within the article.

## References

[1] Ye L., Zhang W.H., Cui Y.C., Deng S.R. (2023). Dynamic Evaluation of the Degradation Process of Vibration Performance for Machine Tool Spindle Bearings. Sensors.

[2] Cai G.G., Chen X.F., Li B., Chen B.J., He Z.J. (2012). Operation Reliability Assessment for Cutting Tools by Applying a Proportional Covariate Model to Condition Monitoring Information. Sensors.

[3] Azmi A.I. (2015). Monitoring of tool wear using measured machining forces and neuro-fuzzy modelling approaches during machining of GFRP composites. Adv. Eng. Softw..

[4] He X.C. (2016). Recent development in reliability analysis of NC machine tools. Int. J. Adv. Manuf. Technol..

[5] Lai X.W., Zhang K., Zheng Q., Li Z.X., Ding G.F., Ding K. (2023). A frequency-spatial hybrid attention mechanism improved tool wear state recognition method guided by structure and process parameters. Measurement.

[6] Kuntoglu M., Aslan A., Pimenov D.Y., Usca Ü., Salur E., Gupta M.K., Mikolajczyk T., Giasin K., Kaplonek W., Sharma S. (2021). A Review of Indirect Tool Condition Monitoring Systems and Decision-Making Methods in Turning: Critical Analysis and Trends. Sensors.

[7] Wang Q.Q., Jin Z.J., Zhao Y., Niu L., Guo J. (2021). A comparative study on tool life and wear of uncoated and coated cutting tools in turning of tungsten heavy alloys. Wear.

[8] Cheng Y.N., Gai X.Y., Guan R., Jin Y.B., Lu M.D., Ding Y. (2023). Tool wear intelligent monitoring techniques in cutting: A review. J. Mech. Sci. Technol..

[9] Bernini L., Malguzzi U., Albertelli P., Monno M. (2024). Hybrid prognostics to estimate cutting inserts remaining useful life based on direct wear observation. Mech. Syst. Signal Process..

[10] Castejón M., Alegre E., Barreiro J., Hernández L.K. (2007). On-line tool wear monitoring using geometric descriptors from digital images. Int. J. Mach. Tools Manuf..

[11] Wang Z.R., Zou Y.F., Zhang F. A Machine Vision Approach to Tool Wear Monitoring Based on the Image of Workpiece Surface Texture. Proceedings of the International Conference on Advances in Materials and Manufacturing Processes.

[12] Niaki F.A., Mears L. (2018). A probabilistic-based study on fused direct and indirect methods for tracking tool flank wear of Rene-108, nickel-based alloy. Proc. Inst. Mech. Eng. Part B J. Eng. Manuf..

[13] Pimenov D.Y., Gupta M.K., da Silva L.R.R., Kiran M., Khanna N., Krolczyk G.M. (2022). Application of measurement systems in tool condition monitoring of Milling: A review of measurement science approach. Measurement.

[14] Ünal P., Deveci B.U., Özbayoglu A.M. A Review: Sensors Used in Tool Wear Monitoring and Prediction. Proceedings of the 18th International Conference on Mobile Web and Intelligent Information Systems (MobiWIS).

[15] Wang J.J., Li Y.L., Zhao R., Gao R.X. (2020). Physics guided neural network for machining tool wear prediction. J. Manuf. Syst..

[16] Xu W.X., Miao H.H., Zhao Z.B., Liu J.X., Sun C., Yan R.Q. (2021). Multi-Scale Convolutional Gated Recurrent Unit Networks for Tool Wear Prediction in Smart Manufacturing. Chin. J. Mech. Eng..

[17] Qin Y.Y., Liu X.L., Yue C.X., Zhao M.W., Wei X.D., Wang L.H. (2023). Tool wear identification and prediction method based on stack sparse self-coding network. J. Manuf. Syst..

[18] Cheng Y.N., Jin Y.B., Gai X.Y., Guan R., Lu M.D. (2023). Prediction of tool wear in milling process based on BP neural network optimized by firefly algorithm. Proc. Inst. Mech. Eng. Part E J. Process Mech. Eng..

[19] Wang C.H., Shen B. (2024). Auxiliary input-enhanced siamese neural network: A robust tool wear prediction framework with improved feature extraction and generalization ability. Mech. Syst. Signal Process..

[20] Kong D.D., Chen Y.J., Li N., Duan C.Q., Lu L.X., Chen D.X. (2019). Relevance vector machine for tool wear prediction. Mech. Syst. Signal Process..

[21] Wang J.Q., Xiang Z., Cheng X., Zhou J., Li W.Q. (2023). Tool Wear State Identification Based on SVM Optimized by the Improved Northern Goshawk Optimization. Sensors.

[22] Kuntoglu M., Saglam H. (2021). Investigation of signal behaviors for sensor fusion with tool condition monitoring system in turning. Measurement.

[23] Buj-Corral I., Sender P., Luis-Pérez C.J. (2023). Multi-objective optimization of tool wear, surface roughness, and material removal rate in finishing honing processes using adaptive neural fuzzy inference systems. Tribol. Int..

[24] Wang J.J., Yan J.X., Li C., Gao R.O.E., Zhao R. (2019). Deep heterogeneous GRU model for predictive analytics in smart manufacturing: Application to tool wear prediction. Comput. Ind..

[25] Shah M.L., Borade H., Sanghavi V., Purohit A., Wankhede V., Vakharia V. (2023). Enhancing Tool Wear Prediction Accuracy Using Walsh-Hadamard Transform, DCGAN and Dragonfly Algorithm-Based Feature Selection. Sensors.

[26] Liu Q., Li D.K., Ma J., Wei X.D., Bai Z.Y. (2023). A multi-input parallel convolutional attention network for tool wear monitoring. Int. J. Comput. Integr. Manuf..

[27] Abdeltawab A., Xi Z., Zhang L.J. (2024). Tool wear classification based on maximal overlap discrete wavelet transform and hybrid deep learning model. Int. J. Adv. Manuf. Technol..

[28] Si X.S., Wang W.B., Hu C.H., Zhou D.H., Pecht M.G. (2012). Remaining Useful Life Estimation Based on a Nonlinear Diffusion Degradation Process. IEEE Trans. Reliab..

[29] Yan B.X., Ma X.B., Huang G.F., Zhao Y. (2021). Two-stage physics-based Wiener process models for online RUL prediction in field vibration data. Mech. Syst. Signal Process..

[30] Chang C.C., Lin C.J. (2011). LIBSVM: A Library for Support Vector Machines. Acm Trans. Intell. Syst. Technol..

[31] Smola A.J., Schölkopf B. (2004). A tutorial on support vector regression. Stat. Comput..

[32] Prognostics and Health Management Society (PHM Society). https://phmsociety.org/phm_competition/2010-phm-society-conference-data-challenge/.

